# Validation of a Loop-Mediated Isothermal Amplification Assay for Rapid Diagnosis of Invasive Pneumococcal Disease

**DOI:** 10.3389/fcimb.2020.00115

**Published:** 2020-03-24

**Authors:** Héctor David de Paz, Pedro Brotons, Cristina Esteva, Carmen Muñoz-Almagro

**Affiliations:** ^1^Department of Molecular Microbiology, Institut de Recerca Pediatrica, Hospital Sant Joan de Déu, Barcelona, Spain; ^2^CIBER of Epidemiology and Public Health, CIBERESP, Madrid, Spain; ^3^Department of Medicine, Universitat Internacional de Catalunya, Barcelona, Spain

**Keywords:** *Streptococcus pneumoniae*, loop-mediated isothermal amplification, invasive pneumococcal disease, rapid diagnosis, diagnostic accuracy

## Abstract

Current molecular PCR-based techniques used for detecting *Streptococcus pneumoniae*, the causative pathogen of invasive pneumococcal disease (IPD), are accurate but have a run time of several hours. We aimed to develop and validate a novel real-time loop mediated amplification (LAMP) assay for rapid detection of pneumococcus in normally sterile samples with accuracy comparable to a gold standard real-time PCR. Conserved regions of *lytA* were used for the design of the LAMP test. Analytical validation included assessment of linearity, limit of detection (LOD), intra-assay and inter-assay precision and analytical specificity, which was evaluated by using reference strain *S. pneumoniae* R6 and a quality control panel. Clinical performance was assessed on all samples collected from children with suspicion of IPD attended in Hospital Sant Joan de Deu (Barcelona, Spain) during the period April-September 2015. Fresh samples were analyzed after DNA extraction. The following values of analytical parameters were determined: linearity within the range 10^8^-10^4^ copies/mL; limit of detection, 5·10^3^ copies/mL; intra- and inter-assay precision measured by mean coefficient of variance, 3.61 and 6.59%; analytical specificity, 9/9 pathogens similar to *S. pneumoniae* and 14/14 strains of different *S. pneumoniae* serotypes correctly identified as negative and positive results, respectively. Diagnostic sensitivity and specificity values were 100.0 and 99.3%. Median time of DNA amplification was 15 min. The new LAMP assay showed to have similar accuracy as PCR while being 5-fold faster and could become a useful diagnostic tool for early diagnosis of IPD.

## Introduction

*Streptococcus pneumoniae* is a potentially pathogenic bacteria of the human nasopharynx (Bogaert et al., 2004) and a major cause of morbidity and mortality worldwide, especially among young children and elderly population (Obaro and Adegbola, 2002). Despite the decrease of invasive pneumococcal disease (IPD) incidence after pneumococcal conjugated vaccines introduction (Yildirim et al., 2012; Harboe et al., 2014; Lai et al., 2014), IPD remains a major global health problem. The World Health Organization estimated that pneumococcus caused 550,000 deaths of children younger than 5 years in 2015 (Walker et al., 2013). Early diagnosis of life-threatening IPD, such as pneumococcal meningitis or bacteremia/sepsis, is essential for prompt and targeted treatment (Dellinger et al., 2013).

Diagnostic techniques such as culture or antigen detection have traditionally been used for microbiological diagnosis of IPD but they are time-consuming and/or lack sensitivity (Fierz, 2004; Bidet et al., 2008). Molecular techniques like polymerase chain reaction (PCR) have improved diagnostic time since onset of symptoms in a sensitive and specific way (Nilsson et al., 2008; Muñoz-Almagro et al., 2011). However, PCR methods require utilization of costly equipment and operation by highly trained technicians. Therefore, PCR-based diagnostic tests do not meet several “ASSURED” criteria for the ideal diagnostic test, namely to be affordable, sensitive, specific, user-friendly, rapid and robust, equipment-free and deliverable to end users (Kosack et al., 2017).

In recent years, the emergence of novel isothermal techniques such as nicking endonuclease amplification reaction (NEAR), strand displacement amplification (SDA), loop-mediated isothermal amplification (LAMP), and helicase-dependent amplification (HAD) has led to the emergence of inexpensive and easy-to-use diagnostic devices that fulfill most ASSURED criteria (de Paz et al., 2014). Particularly, the LAMP technique (Notomi, 2000) has been reported to have similar, or even better, diagnostic accuracy than PCR for detecting infectious agents (Chen et al., 2011; Parida et al., 2011; Patel et al., 2014; Brotons et al., 2016), including *S. pneumoniae* (Seki et al., 2005; Kim et al., 2012). LAMP chemistry is based on the *Bst* DNA polymerase and the simultaneous use of 3 pairs of primers. On one hand, *Bst* polymerase is an enzyme with strand-displacement activity, which synthesizes a new DNA strand while dissociating the double-stranded DNA template itself. On the other hand, the combined use of 6 pairs of primers allows a rapid and very specific amplification. In addition, the *Bst* polymerase has a high tolerance to inhibitory substances commonly present in biological samples. All these features make this molecular chemistry highly suitable for diagnostic use in resource-limited settings (Notomi, 2000; de Paz et al., 2014).

In the present study we developed a real-time fluorescent LAMP assay for *S. pneumoniae* detection in invasive biological samples (pleural fluid, plasma, cerebrospinal fluid, and synovial liquid) and compared its analytical and diagnostic performance against a gold standard real-time PCR assay.

## Materials and Methods

### Study Design and Setting

The study was conducted in the Molecular Microbiology Department of Children's University Hospital Sant Joan de Déu in Barcelona (Spain). This hospital captures around 20% of all pediatric hospitalizations within Catalonia, a region with ~7 million population, 1.2 million of them aged <18 years.

### Sample Collection and DNA Extraction

Biological samples of patients and reference control strains were extracted and concentrated by NucliSENS^®^EasyMag^®^ (bioMérieux Inc., NC, US) from an initial volume of 200 μl to an elution volume of 50 μl.

### LAMP Assay Design

Conserved regions of *lytA* were used as a diagnostic target for *S. pneumoniae* detection. More than 70 sequences of *S. pneumoniae* annotated in PUBMED database were analyzed to obtain the consensus sequence comparing it with *Streptococcus pseudoneumoniae* and *Streptococcus mitis* sequences. Primer Explorer version 4 software (Eiken Chemical Co., Tokyo, Japan) was utilized for primer design. Sequence of *lytA* primers is detailed as follows: F3 (5′GCT GGA AGA AAA TCG CTG A 3′), B3 (5′ GAA TTC TGG CCT GTC TGC), FIP (5′ TCT AAG TAG TAC CAA GTG TCC TTG TGG TAC TAT TTC AAC GAA GAA GGT 3′), BIP (5′ GCT AAA GAA GGC GCC ATG GTC GTC TGG TTT GAG GTA GT 3′), LF (5′ CCC AGC CTG TCT TCA TGG C 3′), LB (5′ ATC AAA TGC CTT TAT CCA GTC AGC 3′).

Five micro-liters of extracted DNA were added to 20 μl of Master Mix ISO-001 (Optigene, UK) with appropriate primer concentrations: F3 (0.2 μM), B3 (0.2 μM), FIP (2 μM), BIP (2 μM), LoopF (0.8 μM), and LoopB (0.8 μM). LAMP assay was performed at 65°C for 30 min using CFX96 real-time PCR Detection System (BioRad Laboratories, CA, US). The time-to-detection value was defined as the time in minutes at the reporting dye fluorescence that first exceeds the calculated background level. A low time-to-detection value thus corresponds to a high target concentration.

### Real-Time PCR Assay

A duplex real-time PCR targeting the *lytA* gene of *S. pneumoniae* and the internal control targeting RNAse P of human cells was performed. Sequence of primers and probes recommended by CDC for both the pathogen and the internal control were used (Centers for Disease Control and Prevention (CDC), 2015). The probe targeting the *lytA* gene was fluorescence-marked in FAM whereas the probe targeting the RNAse P was marked in YAK.

DNA was amplified with the Applied Biosystems 7500 real-time PCR System (Applied Biosystems, CA, US) using the following cycling parameters: 50°C for 2 min and 95°C for 10 min, followed by 45 cycles at 95°C for 15 s and 60°C for 1 min (total time: 1 h and 59 min). Amplification data were analyzed with SDS software (Applied Biosystems, CA, US). The threshold cycle (Ct) value for the PCR assay was defined according to the same criteria set for the time-to-detection value of the LAMP assay.

### Analytical Validation of the LAMP Assay

Analytical validation was performed by using reference strain *S. pneumoniae* R6 and plasma matrices. Given that genome-copies number is less variable than colony forming units (CFU) for making standards (Morpeth et al., 2014), values are presented in genome copy number which is about 2-logs higher than CFU. A calibration curve was generated by extracting the genomic DNA from an original suspension from the *S. pneumoniae R6* strain (OD_595_ = 0.5) and performing 10-fold serial dilutions that ranged from 10^8^ to 10^2^ copies per ml (cp/mL). DNA concentration was estimated in ng/μl using the Quawell Q5000 micro volume spectrophotometer (Quawell, CA, US). The number of genomic copies was determined assuming the molecular size of *S. pneumoniae* R6 (2.04 Mpb) and using the formula mass = DNA size (base pairs) × 1 mole/6.023·10^23^ molecules × 660 g/mole.

Evaluation of the linear range was performed through analysis of 3 replicates at each concentration across the 10^8^-10^3^ cp/mL range. The Limit of Detection (LOD) was determined by testing 20 replicates at two low detectable concentrations (10^3^ and 10^2^ cp/mL) and confirming positive results in ≥95% of all replicates. Precision was assessed by testing 3 replicates in the same run and 5 replicates in different runs, different days and by different operators at the 10^8^-10^4^ cp/mL range to calculate intra- and inter-assay variability, respectively, by means of the coefficient of variance (CV).

A quality control panel was performed with DNA extracts (*n* = 14) from plasma matrices with positive detection of different pneumococcal serotypes at 10^4^ cp/mL concentration. DNA extracts were stored at −80°C in our site collection. Serotype determination was performed as described in previous studies (Selva et al., 2012, 2014). In addition, 10 bacterial strains of different pathogens at 5·10^7^ cp/mL and human DNA at 10^5^ cp/mL were also included in the panel to determine analytical specificity of the LAMP assay. *S. pneumoniae* R6 strain was provided by the Molecular Microbiology and Infection Biology Department of the Biological Research Center (CIB-CSIC, Madrid, Spain). *B. pertussis* CECT7974 and *Bordetella bronchipseptica* ATCC4617 strains were obtained from the Spanish Collection of Cell Cultures (CECT) while *Bordetella parapertussis* ATCC15311 and *Bordetella holmesii* ATCC51541 were obtained from DSMZ-German Collection of Microorganisms and Cell Culture. The rest of bacteria in the panel were retrieved from our clinical laboratory collection.

### Diagnostic Validation of the LAMP Assay

All normally sterile samples collected from children <18 years suspected of IPD and attended in the study site between April 2015 and September 2015 were included in the diagnostic validation study. Routine analysis by LAMP and PCR was prospectively performed with fresh samples suspected to contain pneumococcus. Blood culture was also performed in all samples and used to resolve discrepancies between LAMP and PCR results. Sensitivity and specificity values were determined as reported elsewhere (Hess et al., 2012). In addition, DNA amplification times by both PCR and LAMP were recorded to assess rapidity of the techniques.

### Ethical Considerations

All data were duly anonymized. Only routine microbiological results were available for comparison of results. No clinical or epidemiological data of patients were registered. The study was approved by the Ethics Committee of the hospital setting.

### Statistical Analysis

Statistical analysis was performed with Statistical Package for Social Sciences SPSS software (IBM Corp., US). After log_10_ transformation linear regression analysis was performed to define the span of result values across the concentration range. LOD was calculated by probit regression analysis. Precision was evaluated by obtaining mean time-to-detection values and standard deviations (SD) of each set of replicates at a given concentration and calculating coefficients of variation (CV = SD/Mean). Dichotomous positive and negative results by the LAMP and PCR assays were analyzed using a 2 × 2 contingency table. Confidence Intervals (CI) were set at 95% and significance at a 2-sided *p*-value of <0.05 for all statistical analysis.

## Results

### Analytical Performance

The LAMP assay was able to detect *S. pneumoniae* DNA over a linear range of 10-fold serial dilutions between 10^8^ and 10^4^ cp/mL (r^2^ = 0.9152; y = 0,9677x + 13.344) while real-time PCR showed a linear range between 10^8^ and 10^3^ cp/mL (r^2^ = 0.9999; y = −3.3851x + 48.038). A LOD value of 5·10^3^ cp/mL (90 copies per reaction; cp/rx) was determined for the LAMP test, not far from the LOD value of 1·10^3^ cp/mL (20 cp/rx) observed for PCR.

Among the 14 *S. pneumoniae* DNA extracts from plasma samples included in the quality control panel all of them yielded positive results for both PCR and LAMP. On the other hand, no cross-reactivity was found either with other bacteria or with human DNA (Table 1).

**Table 1:** Quality control panel performed with DNA extracts from direct clinical samples and bacterial strains.

**DNA extract**	**Source**	**Result by LAMP**	**Result by PCR**
*S. pneumoniae* serotypes			
1	Pleural fluid	+	+
3	Plasma	+	+
3 and 8^*^	Nasal aspirate	+	+
9N/L ^#^	Nasal aspirate	+	+
10A	Nasal aspirate	+	+
13	cerebrospinal fluid	+	+
14	Plasma	+	+
15B/C^#^	Nasal aspirate	+	+
19A	Nasal aspirate	+	+
21	Nasal aspirate	+	+
23B	Pleural fluid	+	+
24	Nasal aspirate	+	+
33F/A/37^#^	Nasal aspirate	+	+
*S. pneumoniae* (R6)	CIB-CSIC	+	+
*B. pertussis*	CECT7974	-	-
*B. parapertussis*	ATCC15311	-	-
*B. holmesii*	ATCC51541	-	-
*B. bronchiseptica*	ATCC4617	-	-
*N. meningitidis*	Own collection	-	-
*S. aureus*	Own collection	-	-
*E. coli*	Own collection	-	-
*S. mitis*	Own collection	-	-
*S. pseudopneumoniae*	Own collection	-	-

*+, positive. -, negative*.

* *Co-colonization*.

# *Serotypes not distinguishable by performed methods*.

LAMP intra- and inter-assay mean CV values across the linear range were 3.61 and 6.59%, respectively, as shown in Tables 2, 3. Between-run CV values ranged from 0.16% at 10^6^ cp/mL to 5.68% at 10^8^ cp/mL. Within-run CV values were higher at all concentrations tested, ranging from 6.23% at 10^6^ cp/mL to 8.89% at 10^8^ cp/mL.

**Table 2:** Results of precision (intra-assay variability) for the LAMP assay.

**Bacterial load (cp/mL)**	**No. replicates**	**Mean T-t-D(min)**	**SD**	**CV (%)**
10^8^	3	5.28	0.30	5.68
10^7^	3	6.89	0.38	5.49
10^6^	3	7.13	0.01	0.16
10^5^	3	7.93	0.20	2.51
10^4^	3	9.60	0.40	4.19
			Mean CV (%)	3.61

*Cp, genomic copies. T-t-D, time-to-detection. SD, standard deviation. CV, coefficient of variation*.

**Table 3:** Results of precision (inter-assay variability) for the LAMP assay.

**Bacterial load (cp/ml)**	**No. replicates**	**Mean T-t-D (min)**	**SD**	**CV (%)**
10^8^	5	5.34	0.47	8.89
10^7^	5	6.73	0.44	6.52
10^6^	5	7.41	0.46	6.23
10^5^	5	8.32	0.55	6.58
10^4^	5	10.00	0.70	7.03
			Mean CV (%)	6.59

*Cp, genomic copies. T-t-D, time-to-detection. SD, standard deviation. CV, coefficient of variation*.

### Diagnostic Performance

During the study period, a total of 161 normally sterile samples (124 plasma fluids, 13 cerebrospinal fluids, 13 pleural fluids, and 11 synovial liquids) were collected. Thirteen (8.07%) were detected as positive by LAMP and 12 (7.45%) as negative by real-time PCR (Table 4) whereas there were 148 negatives by LAMP and 149 negatives by PCR. Diagnostic sensitivity and specificity of the LAMP assay compared to real-time PCR were 100% (95% CI, 75.8–100) and 99.3% (95% CI, 97.5–100), respectively. The discrepant sample, which corresponded to a pleural fluid, was repeated by both real-time PCR and culture and yielded a PCR-positive result and a negative result by culture. Of note, all positive results by both LAMP and PCR tests were negative by culture whereas culture yielded 17 positive results for bacteria different from pneumococcus that were negative by the two molecular methods. Specifically, bacteria identified by culture included *Staphylococcus aureus* (*n* = 6), *Neisseria meningitidis* (*n* = 3), *Streptococcus viridans*(*n* = 3), *Kingella kingae* (*n* = 2), *Micrococcus* spp. (*n* = 1), and *Propionibcaterium acnes* (*n* = 1). Positive PCR, LAMP, and culture results per type of specimen are detailed in Supplementary Tables 1, 2.

**Table 4:** Comparison of diagnostic results by LAMP vs. real-time PCR.

	**Reference PCR test**		
LAMP test	**Positive**	**Negative**	**Total**
Positive	12	1	13
Negative	0	148	148
**Total**	12	149	161

Pneumococcal DNA was amplified by LAMP within 30 min (median time: 15 min) while PCR amplification needed a maximum time of 103 min (median time: 79 min), which represents a median 5-fold decrease in time for DNA amplification.

## Discussion

In the current study we developed a real-time LAMP assay targeting *lytA* gene, for *S. pneumoniae* detection and assessed analytical and diagnostic performance of the test against a reference real-time PCR. Since both *lytA* and *ply* targets have similar sensitivity but *lytA* gene contributes higher specificity, the latter was selected as the target gene of our LAMP reaction.

LOD of the LAMP assay (5·10^3^ cp/mL 90 cp/rx) was found to be slightly higher though in the same order of magnitude as LOD of PCR (1·10^3^ cp/mL, 20 cp/rx). These values are in contrast with values of analytical sensitivity reported to be 3-logs higher for LAMP than for conventional PCR in previous studies measuring LOD of PCR at around 10^4^ cp/rx (Seki et al., 2005; Huy et al., 2012; Kim et al., 2012). Differences in PCR analytical sensitivity between studies could be due to the selected way of amplified DNA visualization (electrophoretic analysis). In this regard, it should be noted that we compared the LAMP assay with a real-time PCR with TaqMan probes. In fact other studies reported LOD values for PCR around 50 cp/rx (Nandakumar et al., 2008) and 22 cp/rx (Sheppard, 2004), which are in line with the LOD of 20 cp/rx obtained for the reference PCR of our study.

Other LAMP techniques targeting the *lytA* gene have determined similar values of analytical sensitivity (10 cp/rx) as those described our assay (Seki et al., 2005; Kim et al., 2012). Minor differences between LAMP sensitivities could be explained by the higher number of replicates that we used for LOD calculation, as suggested by guidelines (FDA, 2001), in comparison to the number of replicates considered in the referenced studies (<3). The LOD described in our study is also in concordance with the value of 100–500 CFU/mL (between 10^3^ and 10^4^ cp/mL) targeting the 16S rRNA gene (Huy et al., 2012) and the *ply* gene (Wang et al., 2018). Conversely, another published work established a LOD about 300 pg/μL (10^5^ copies/μl) for a specific LAMP test based on SYBR Green fluorophore and using a portable tube scanner (Xia et al., 2014).

The new LAMP assay was able to detect specifically all the 14 *S. pneumoniae* serotypes tested. In addition, no cross-reactivity was detected either with human DNA or with the rest of tested bacteria, not even with genetically similar bacteria such as *S. mitis* or *S. pseudoneumoniae*. The use of *lytA* gene as the amplification target and the fact that LAMP reaction occurs only when six regions within a target DNA are recognized (Notomi, 2000) contributed high specificity to the new assay. Although multiple strains of *S. pneumoniae* and other pathogens and commensal bacteria of the nasopharynx have been tested, the modest size of the quality control panel might be a limitation of the study.

Intra- and inter-assay precision of the new test across the range of expected concentrations showed to be adequate. Mean CV values obtained did not exceed the recommended value of 15% for imprecision (FDA, 2001). Of note, linearity (10^8^-10^4^ cp/mL) was lower than that of PCR (10^8^-10^3^ cp/mL), which may indicate better suitability of the test for qualitative or semi-quantitative diagnosis than for quantification.

LAMP diagnostic sensitivity (100%) and specificity (99.3%) were optimal. Only a discrepant result was observed and after confirmatory PCR classified as a true positive result. The related samples showed a bacterial load under the LOD (Ct 39, 5; about 4 cp/rx) which could explain the initial discordance.

In addition, the LAMP assay proved to be a rapid method for detection of pneumococcus, allowing for a 5-fold median run time reduction in comparison with PCR. It should be noted that total time required for PCR amplification could be reduced with cycle alterations slightly below 1 h. However, we followed the PCR protocol recommended by the CDC to ensure adequate sensitivity and specificity while achieving much faster time of amplification with LAMP. The LAMP reaction is a single tube technique for DNA amplification that does not require thermal cyclers (Huy et al., 2012; Xia et al., 2014; Wang et al., 2018). In addition, amplification can be detected by turbidimetry or visual color change without the need for any other equipment (Huy et al., 2012). Even electricity-free and reusable devices heaters have been developed for DNA amplification using the LAMP technique (Sema et al., 2015). All these characteristics, together with LAMP high sensitivity and specificity, favor the use of this technique as a simple and inexpensive diagnostic assay, which would be especially useful in low and middle income countries (Geojith et al., 2011). In this sense, it should be mentioned that the LAMP assay described in this study could be integrated into a prototype of a rapid, simple, and cost-effective point-of-care detection of different respiratory pathogens including *S. pneumoniae*.

We performed a real-time PCR assay in samples collected from normally sterile sites, the majority of which were blood specimens, and considered this assay as a reference standard for validating the LAMP test. Pneumococcal real-time PCR in blood is widely used in hospital environments for routine diagnostics of pneumococcal infections and the European Union regulations have stated the adequacy of pneumococcal DNA detection for confirmation of cases with pneumococcal infection or IPD in normally sterile samples (Commission Implementing Decision EU 2018/945, 2018). However, there is some controversy about the consideration of blood pneumococcal PCR as a gold standard, since diverse studies have reported noticeable proportions of blood pneumococcal PCR-positive results in healthy children (Morpeth et al., 2017) and moderate values of sensitivity and specificity of PCR in blood samples extracted from children with IPD (Avni et al., 2010).

In conclusion, we designed and validated a real-time LAMP assay based on fluorescence for diagnosis of IPD. The test proved to be 5-fold faster than PCR for DNA amplification while maintaining similar levels of analytical and diagnostic performance. This novel LAMP assay could be useful for early detection of *S. pneumoniae*, especially if integrated into inexpensive easy-to-use point-of-care diagnostic devices not requiring the complex electronics associated to cyclic temperature changes.

## Data Availability Statement

The datasets generated for this study are available on request to the corresponding author.

## Author Contributions

CE performed the laboratory experiments. HP organized the database and wrote the first draft of the manuscript. PB performed the statistical analysis. HP and PB wrote sections of the manuscript. HP, PB, and CM-A contributed conception and design of the study. All authors contributed to manuscript revision, read and approved the submitted version.

### Conflict of Interest

CM-A reports research grants to her institution from Pfizer, BioFire Diagnostics, Roche Diagnostics, BioMérieux, Alere and Genomica SAU, and compensation fees from Qiagen, Roche Diagnostics and BioMérieux for scientific presentations in satellite symposiums outside the submitted work. PB reports compensation fees from Roche Diagnostics for scientific presentations in satellite symposiums outside the submitted work. The remaining authors declare that the research was conducted in the absence of any commercial or financial relationships that could be construed as a potential conflict of interest.
