# Supplementary material for: Validation of a Loop-Mediated Isothermal Amplification Assay for Rapid Diagnosis of Invasive Pneumococcal Disease
Source: Front Cell Infect Microbiol. 2020 Mar 24;10:115. doi: 10.3389/fcimb.2020.00115 (PMC7105778; doi:10.3389/fcimb.2020.00115)
Supplement: Supplementary file 1 [file Data_Sheet_1.docx]

**Supplementary Table 1. Positive PCR and LAMP results per type of specimen**

| **Specimen type** | **Total samples** | **Positives by PCR** | **Positives by LAMP** |
| --- | --- | --- | --- |
| pleural fluid | **13** | **5** | **5** |
| plasma | **123** | **5** | **6** |
| cerebrospinal fluid | **13** | **1** | **1** |
| synovial liquid | **11** | **1** | **1** |
| pericardial fluid | **1** | **0** | **0** |
| Total | **161** | **12** | **13** |

**Supplementary Table 2. Positive PCR, LAMP and culture results per type of specimen**

| **Specimen type** | **Total samples** | **Positives by PCR** | **Positives by LAMP** | **Positives by Culture** |
| --- | --- | --- | --- | --- |
| pleural fluid | **12** | **4** | **4** | **0** |
| plasma | **89** | **5** | **6** | **7** |
| cerebrospinal fluid | **13** | **1** | **1** | **1** |
| synovial liquid | **9** | **1** | **1** | **1** |
| pericardial fluid | **1** | **0** | **0** | **0** |
| Total | **124** | **11** | **12** | **9** |
